# Comparison of Paramedian Versus Midline Extraction Sites in Elective Laparoscopic Right Colectomy: A Propensity-Matched Study of Postoperative Ventral Hernia Development

**DOI:** 10.3390/jcm14155198

**Published:** 2025-07-22

**Authors:** Fahim Kanani, Naheel Mahajna, Wasim Shaqqur, Anastasiia Iserlis, Chaled Alnakib, Mordechai Shimonov, Amir Nutman, Alaa Zahalka, Nir Messer, Arkadiy Iskhakov, Moshe Kamar, Katia Dayan

**Affiliations:** 1 Department of Surgery, Wolfson Medical Center, Holon 58100, Israel, affiliated with the Gray Faculty of Medicine and Science, Tel Aviv University, Tel Aviv, Israel; naheelmahajna@gmail.com (N.M.); waseem.shakkour@gmail.com (W.S.); chaled88aw@gmail.com (C.A.); shimonov.m@wmc.gov.il (M.S.); alaa.zahalka@hotmail.co.il (A.Z.); masrinir@gmail.com (N.M.); doc_arkadi@walla.co.il (A.I.); dr.kamar.moshe@gmail.com (M.K.); katiad@wmc.gov.il (K.D.); 2 Hospital Management, Wolfson Medical Center, Holon 58100, Israel, affiliated with the Gray Faculty of Medicine and Science, Tel Aviv University, Tel Aviv, Israel; nastyayasko23@gmail.com (A.I.); amirnu@wmc.gov.il (A.N.)

**Keywords:** postoperative ventral hernia, laparoscopic colectomy, extraction site, propensity matching, risk factors

## Abstract

**Background**: Postoperative ventral hernia (POVH) remains a significant complication following laparoscopic colectomy despite minimally invasive approaches. Extraction site selection may influence POVH incidence, yet optimal location remains controversial. **Methods**: This retrospective cohort study analyzed 550 patients undergoing elective laparoscopic right colectomy (2009–2024) at a single center. After exclusions for anastomotic leak and loss to follow-up, 266 patients were propensity-matched 1:1 comparing paramedian (n = 133) versus midline (n = 133) extraction sites. The primary outcome was POVH incidence at 36 months. Secondary outcomes included risk factor identification using multivariate logistic regression and Firth penalized methods. **Results**: POVH occurred in 3/133 (2.3%) paramedian versus 15/133 (11.3%) midline patients (*p* = 0.007). Multivariate analysis identified midline extraction (aOR 30.3, 95% CI: 3.34–969, *p* < 0.001), chronic cough (aOR 25.6, 95% CI: 3.56–287, *p* = 0.001), and constipation (aOR 10.1, 95% CI: 1.60–70.7, *p* = 0.015) as independent POVH predictors. Patient comorbidities showed stronger associations than surgical factors in univariate analysis. The number needed to treat with paramedian extraction to prevent one POVH was 11.1. **Conclusions**: Paramedian extraction sites significantly reduce POVH incidence compared to midline approaches in laparoscopic right colectomy. The identification of modifiable physiological risk factors, particularly conditions causing increased intra-abdominal pressure (chronic cough, constipation), suggests that comprehensive perioperative optimization targeting these specific factors may further reduce POVH risk.

## 1. Introduction

Postoperative ventral hernia (POVH) represents a major burden following laparoscopic colectomy, with reported incidence of 10–25%, that significantly impacts quality of life and healthcare costs [1]. While minimally invasive techniques reduce surgical trauma, the necessary specimen extraction incision remains a vulnerable site for hernia development [1,2,3,4].

Current evidence reveals conflicting data regarding optimal extraction site selection. A 2023 meta-analysis of 25 studies (n = 10,362) reported that transverse incisions were associated with significantly lower risk of incisional hernia compared to midline incisions [5], yet a systematic review of minimally invasive colorectal surgery reported extraction site incisional hernia rates ranging from 2.1% for Pfannenstiel to 16.0% for midline sites [6]. This variability reflects differences in study populations, follow-up protocols, and surgical techniques, with midline incisions remaining the predominant approach despite higher complication rates.

The scarcity of high-quality propensity-matched studies specific to laparoscopic right colectomy represents a critical knowledge gap. Most existing literature combines various colorectal procedures, extraction sites, and patient populations without adequate risk adjustment [7]. The paramedian approach offers theoretical advantages through preservation of linea alba integrity and maintained neurovascular supply but lacks robust comparative data against midline extraction in standardized populations [8].

Furthermore, contemporary understanding of POVH pathophysiology suggests multifactorial etiology involving both technical factors and patient-specific physiologic contributors, particularly conditions affecting intra-abdominal pressure [9]. Comprehensive risk stratification incorporating these variables remains underdeveloped.

This study addresses these limitations by analyzing 15 years of standardized laparoscopic right colectomy data using rigorous propensity matching. Our primary objective was to compare POVH incidence between paramedian and midline extraction sites. Secondary objectives included the identification of modifiable risk factors and development of a risk stratification model to guide surgical decision-making and perioperative optimization strategies.

## 2. Materials and Methods

### 2.1. Study Design and Population

This retrospective cohort study analyzed prospectively maintained data from Wolfson Medical Center spanning 15 years (January 2009 to December 2024). The institutional review board approved the study protocol (IRB #WOMC-003-25) with waiver of informed consent. All telephone follow-up assessments were conducted as part of our institution’s standard postoperative surveillance protocol for colorectal surgery patients and documented in the electronic medical record. This study retrospectively analyzed these existing clinical data.

We identified 700 consecutive adult patients (≥18 years) undergoing elective laparoscopic right colectomy. Exclusion criteria included emergency surgery, anastomotic leak, incomplete follow-up data, and inability to establish telephone contact for outcome assessment. This strict inclusion criteria prioritized internal validity over generalizability to high-risk populations. After exclusions, 550 patients remained for analysis, with assignment to paramedian (n = 200) or midline extraction site (n = 350) groups based on surgeon preference.

### 2.2. Data Collection

Comprehensive data were prospectively collected and categorized into three domains.

Patient Demographics and Comorbidities: Demographics included age, sex, BMI, and obesity (BMI ≥ 30 kg/m^2^). Conditions associated with increased intra-abdominal pressure were recorded: nocturia, constipation, chronic cough, ascites, and pregnancy/delivery history. Medical comorbidities comprised ASA ≥ 3, hypertension, chronic renal failure, liver disease, diabetes mellitus, ischemic heart disease, pulmonary disease, and dyslipidemia. Medications (steroids, novel oral anticoagulants), laboratory values (hemoglobin, preoperative creatinine, albumin/protein ratio), surgical history, and smoking status were documented.

Operative Characteristics: Surgical approach (laparoscopic vs. open conversion), extraction site details (type, orientation, length), technical factors (Alexis wound protector use, fascial closure technique, suture material), operative metrics (duration, ICU days), and postoperative complications post-colectomy, assessed using Clavien–Dindo classification, were recorded.

POVH Characteristics: Hernia occurrence timing (6–12, 12–24, 24–36 months), detection method (HerQLes questionnaire, clinical examination confirmed by diagnostic means, incidental CT findings), management requirements, and quality of life impact were assessed. Hernias were classified according to the European Hernia Society (EHS) classification system for primary and incisional abdominal wall hernias, specifically using location (M1–M5, L1-L4) and width (W1–W3) parameters.

### 2.3. Hernia Assessment Protocol

This retrospective cohort study analyzed existing clinical data supplemented by additional telephone interviews for patients with incomplete follow-up. All telephone study-specific assessments collected information about past events. Verbal consent was obtained for study-specific calls as per IRB approval.

POVH was defined as any fascial defect at the extraction site detected clinically or radiologically. Multi-modal detection included the following:

1. Commonly Used Screening Questions and Associated Studies: questions are presented in Appendix A.
  For suspected hernias, we administered two supplementary questionnaires:
  1.1 Abdominal Hernia-Q (AHQ): A validated tool for assessing patient-reported outcomes in ventral hernia patients, including symptoms like bulging and pain. This is a validated survey administered at predetermined intervals to capture patient-reported symptoms.
  1.2 HerQLes Questionnaire: Development and Validation of a Hernia-Related Quality-of-Life Survey (HerQLes).
2. Clinical Evaluation: Symptomatic patients underwent standardized examination with CT confirmation when indicated.
3. Imaging Review: Routine oncological surveillance scans reviewed for incidental findings.

### 2.4. Surgical Technique

All procedures were performed by board-certified colorectal surgeons using standardized laparoscopic techniques. Following specimen mobilization, extraction sites were created according to surgeon preference. For paramedian extraction, incisions were placed 2–3 cm lateral to the rectus muscle edge, with either transverse orientation (superior to anterior superior iliac spine) or vertical orientation. Midline extractions utilized vertical incisions through the linea alba, either periumbilical or infraumbilical based on tumor location.

Fascial closure followed institutional protocol: continuous mass closure using 1-0 PDS (Ethicon, Inc.) loop sutures with attempted 4:1 suture-to-wound length ratio. While the STITCH trial demonstrating benefits of small-bites technique was published during our study period (2015), formal implementation of 5 mm tissue bites at 5 mm intervals was not standardized across surgeons. All fascial layers were closed in a single layer for midline incisions and separately for paramedian incisions (posterior sheath, anterior sheath). Skin closure was performed with staples (67.7%) or absorbable sutures (32.3%) per surgeon preference.

### 2.5. Statistical Analysis

Propensity score matching (1:1) using a nearest neighbor algorithm with 0.1 caliper width balanced baseline characteristics. Matching followed current reporting guidelines [10] and included age, sex, BMI, ASA classification, hypertension, diabetes, coronary artery disease, chronic renal failure, liver disease, COPD, smoking history, chronic cough, constipation, nocturia/BPH, previous abdominal surgery, and previous hernia repair. Balance was assessed using standardized mean differences < 0.1. The propensity score model achieved a C-statistic of 0.742.

Univariate analysis identified potential risk factors (χ^2^ test for categorical, *t*-test for continuous variables). Variables with *p* < 0.20 entered multivariate logistic regression models. Given the limited number of events (n = 18), we performed Firth penalized regression to address small-sample bias and quasi-complete separation. Time-to-event analysis employed Kaplan–Meier methodology with log-rank testing. Proportional hazards assumptions were verified using Schoenfeld residuals.

Sensitivity analyses were considered but deemed unnecessary given our strict inclusion criteria. Model discrimination was assessed using C-statistic and calibration with Hosmer–Lemeshow testing where applicable.

Statistical significance was set at *p* < 0.05. Analyses used R version 4.1.0 with ‘MatchIt’, logistf’, and ‘survival’ packages.

## 3. Results

### 3.1. Study Population

**Figure 1:**
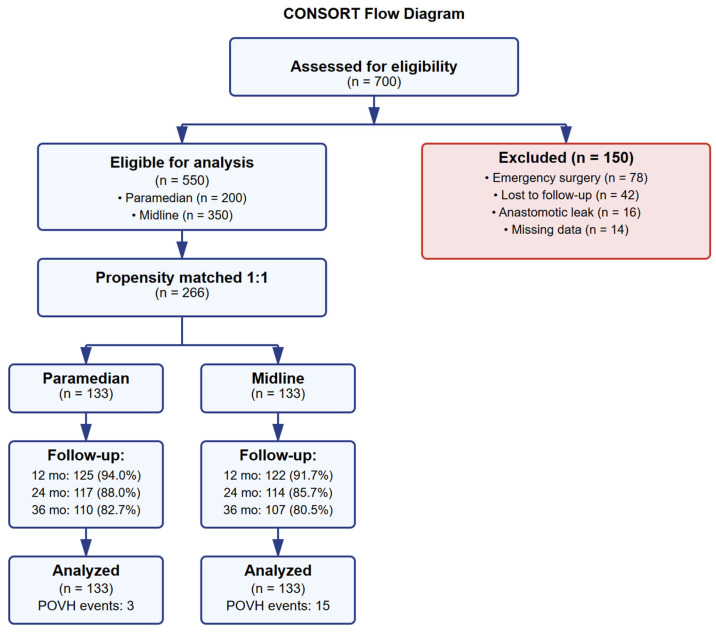
CONSORT flow diagram.

Of 700 patients, 550 met inclusion criteria after excluding 150 patients (78 emergency surgeries, 42 lost to follow-up, 16 anastomotic leaks, 14 missing data) (Figure 1). Before matching, significant baseline differences existed between paramedian (n = 200) and midline (n = 350) groups, with paramedian patients being younger (65.2 vs. 71.4 years, *p* < 0.001) and having lower comorbidity burden (ASA ≥ 3: 32.0% vs. 48.0%, *p* < 0.001) (Table 1 and Supplementary Table S1).

The baseline characteristics after matching propensity matching yielded 266 patients (133 per group) with adequate balance across all variables (all SMD < 0.1). Mean age was 69.0 ± 11.8 years, 51.5% were female, and mean BMI was 27.1 ± 4.4 kg/m^2^. Comorbidities were equally distributed: ASA ≥ 3 (41.4%), hypertension (62.4%), diabetes (28.6%), chronic cough (7.9%), constipation (10.9%), and nocturia (13.2%) (Table 2).

Across the operative characteristics, the laparoscopic completion rates were high (97.7% paramedian vs. 95.5% midline, *p* = 0.498). Paramedian incisions were significantly shorter (5.2 ± 1.3 vs. 6.8 ± 1.8 cm, *p* < 0.001). Technical factors showed no significant differences: wound protector use (100% vs. 97.7%), continuous suture technique (100% both), and PDS (Ethicon, Inc.) suture material (100% vs. 97.7%). Postoperative complications were similar between groups (Clavien–Dindo Grade 0: 94.0% vs. 91.0%, *p* = 0.779) (Table 3).

### 3.2. Follow-Up Completeness

All 266 propensity-matched patients were followed for 12–36 months. Follow-up completeness at 12 months was 94.0% for paramedian and 91.7% for midline groups, at 24 months was 88.0% and 85.7%, and at 36 months was 82.7% and 80.5%, respectively. The weighted median follow-up was 23.5 months for both groups. The mean follow-up duration was 34.5 ± 12.8 months for paramedian and 35.1 ± 13.2 months for midline groups (*p* = 0.707) (Supplementary Table S2).

### 3.3. Primary Outcome and Postoperative Complications

POVH occurred in 3/133 (2.3%) paramedian versus 15/133 (11.3%) midline patients (*p* = 0.007), representing a 79.6% relative risk reduction. The number needed to treat was 11.1 (95% CI: 6.8–28.4). Median time to POVH diagnosis was 22 months overall (IQR 14–28). Time to diagnosis differed by extraction site; paramedian hernias presented at 18, 20, and 28 months, while midline hernias showed bimodal distribution with early (6–12 months, n = 4) and late (24–36 months, n = 8) peaks. Cox proportional hazards analysis confirmed extraction site as the strongest predictor (HR 5.28, 95% CI: 1.52–18.3). The cumulative hernia incidence at 36 months was 2.3% (95% CI: 0.5–6.6%) for paramedian versus 11.3% (95% CI: 6.4–17.9%) for midline extraction (Table 4).

Postoperative complications were comparable between groups. Major complications (Clavien–Dindo ≥ III) occurred in three (2.3%) paramedian versus four (3.0%) midline patients (*p* = 1.000). Surgical site infection rates were similar (4 [3.0%] vs. 5 [3.8%], *p* = 1.000), as were ileus rates (2 [1.5%] vs. 4 [3.0%], *p* = 0.680). Postoperative pneumonia occurred in one patient (0.8%) in each group. No patients developed ARDS. ICU utilization was minimal, with mean ICU days of 0.00 ± 0.00 versus 0.12 ± 1.01 (*p* = 0.170). Median length of stay was 5.2 ± 2.1 days for paramedian and 5.4 ± 2.3 days for midline groups (*p* = 0.462). Thirty-day readmission rates showed no significant difference (11 [8.3%] vs. 13 [9.8%], *p* = 0.668). No 30-day mortality occurred in either group (Table 4).

### 3.4. Risk Factor Analysis

Univariate analysis revealed strong associations with POVH for midline extraction (OR 5.51, *p* = 0.008), open conversion (OR 40.8, *p* < 0.001), constipation (OR 47.1, *p* < 0.001), chronic cough (OR 33.9, *p* < 0.001), nocturia (OR 25.7, *p* < 0.001), smoking (OR 8.22, *p* < 0.001), chronic renal failure (OR 11.4, *p* = 0.006), and liver disease (OR 9.47, *p* = 0.041) (Table 5).

**Figure 2:**
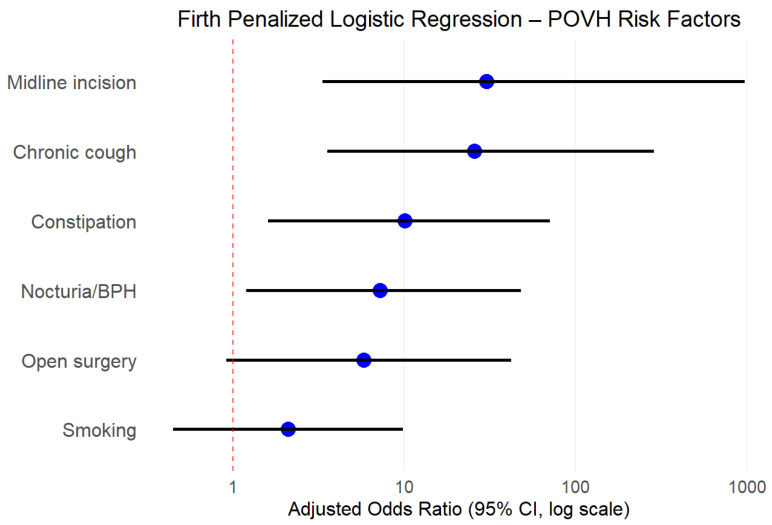
Firth penalized logistic regression—POVH risk factors.

In multivariate Firth penalized regression, three independent predictors emerged: midline extraction (aOR 30.3, 95% CI: 3.34–969, *p* < 0.001), chronic cough (aOR 25.6, 95% CI: 3.56–287, *p* = 0.001), and constipation (aOR 10.1, 95% CI: 1.60–70.7, *p* = 0.015). Model discrimination was excellent (C-statistic = 0.941) (Table 6, Figure 2).

**Figure 3:**
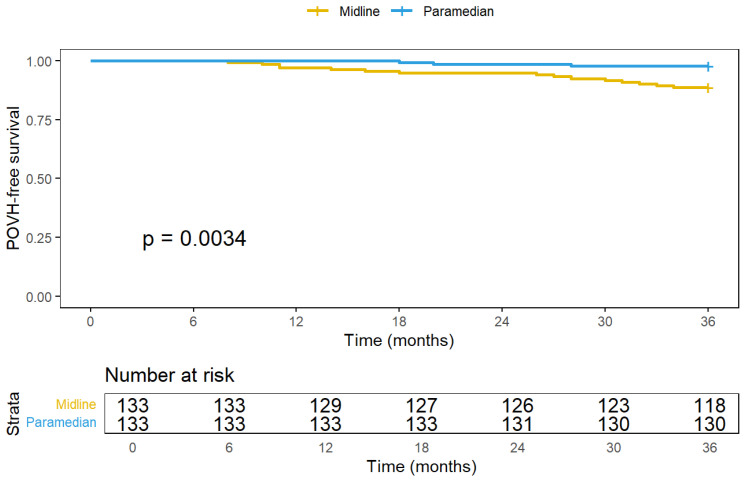
Event-free analysis and Kaplan–Meier analysis.

Event-free analysis and Kaplan–Meier analysis demonstrated a superior POVH-free period for paramedian extraction (log-rank *p* = 0.004, HR 5.28, 95% CI: 1.52–18.3): at 12 months, 100% vs. 97.0%; at 24 months, 98.5% vs. 94.7%; at 36 months, 97.7% vs. 88.7%. Median time to POVH was 22 months (IQR: 14–28) (Figure 3).

Risk stratification showed that high-risk patients (≥2 risk factors: midline extraction, chronic cough, constipation) demonstrated 38.5% POVH incidence versus 1.2% in low-risk patients (0–1 factors), yielding 32.1-fold increased odds (95% CI: 8.9–115.7, *p* < 0.001).

## 4. Discussion

This propensity-matched cohort study demonstrates that paramedian extraction sites reduce postoperative ventral hernia (POVH) incidence compared to midline approaches following laparoscopic right colectomy. The observed 79.6% relative risk reduction (2.3% vs. 11.3%, *p* = 0.007) represents a clinically meaningful difference with important implications for surgical practice. We used propensity score matching to minimize selection bias, achieving standardized mean differences <0.1 [10], and applied Firth penalized logistic regression to address small-sample bias, given our 6.8% event rate.

The anatomical rationale for this difference is compelling. Midline incisions violate the linea alba—a relatively avascular fusion of anterior and posterior rectus sheaths with limited regenerative capacity [6]. Histological studies demonstrate 30% fewer fibroblasts and 40% less vascular density in the linea alba compared to lateral fascial planes [11]. Paramedian incisions preserve this vulnerable midline structure while traversing the well-vascularized rectus muscle, which maintains blood supply from epigastric vessels. The muscle’s contractile properties create dynamic compression across healing fascial edges, promoting primary intention healing [12]. Recent biomechanical modeling demonstrates that midline incisions experience 2.7-fold greater tensile stress during Valsalva maneuvers compared to paramedian sites [13,14], explaining our observed temporal hernia distribution with midline failures occurring throughout follow-up while paramedian sites remained stable.

Our findings align with accumulating evidence supporting off-midline extraction. Den Hartog’s systematic review and meta-analysis of 11,788 patients reported pooled extraction site incisional hernia rates of 16.0% for midline versus 2.1% for Pfannenstiel approaches, with midline extraction showing significantly higher odds of hernia formation compared to non-midline sites (OR 3.2, 95% CI: 2.0–5.3) [6]. Our results may reflect standardized hernia detection protocols and specific advantages of paramedian technique. Benlice’s analysis of 2,148 laparoscopic colorectal resections found extraction-site-specific incisional hernia rates: infraumbilical midline 9.4%, stoma site/right or left lower quadrant 8.6%, periumbilical midline 12.6%, Pfannenstiel 0.9%, and midline converted 12.0% [7]. Our 2.3% paramedian rate aligns closely, supporting external validity. While the European Hernia Society acknowledges extraction site as a modifiable risk factor, they stop short of recommending routine off-midline extraction, citing insufficient evidence [15], a gap our study helps address.

An important finding involves the profound influence of intra-abdominal pressure (IAP) related conditions. Our observed associations—chronic cough (OR 33.9), constipation (OR 47.1), and nocturia/BPH (OR 25.7)—substantially exceed those previously reported in the literature. While prior studies found modest associations (OR 1.4–2.4), our comprehensive detection methods and longer follow-up likely captured the true magnitude of these effects [5,16,17,18]. This suggests current prevention strategies inadequately address physiologic contributors to hernia formation. The pathophysiology involves chronic IAP elevation triggering matrix metalloproteinase activation and accelerating fascial degradation, as demonstrated by Henriksen’s work showing three-fold elevated MMP levels in hernia patients [18].

The combined effect of high-risk surgical approaches with uncontrolled IAP conditions emphasizes perioperative optimization importance. Our risk stratification model, identifying 38.5% POVH incidence in patients with ≥2 risk factors, enables targeted interventions. The economic impact is substantial, with ventral hernia repairs costing USD 3.2 billion annually in the US, and each 1% reduction in hernia recurrence potentially saving USD 32 million [19].

Technical factors also merit consideration. Our universal wound protector use aligns with evidence showing substantial SSI reduction with dual-ring retractors, indirectly supporting hernia prevention [20]. Continuous suturing in both groups reflects current evidence showing equivalent hernia rates versus interrupted techniques [21]. Our predominant PDS (Ethicon, Inc.) use follows recommendations based on reduced inflammatory response compared to rapidly absorbable sutures, though five midline patients receiving Vicryl (Ethicon, Inc.) may have contributed to higher POVH rates [22].

Implementation experiences from other centers support the effectiveness of off-midline approaches, with institutions reporting substantial POVH reductions after transitioning from midline to alternative extraction sites [23,24]. These real-world outcomes demonstrate feasibility and effectiveness of off-midline approaches.

Concerns about lateral hernia repair complexity warrant balanced consideration. The literature indicates that lateral hernias may require advanced techniques like transversus abdominis release more frequently than midline defects [25]. However, recent series report favorable outcomes for paramedian repairs, with only 2.7% incidence and low morbidity when mesh is utilized [9]. Modified techniques for semilunar line hernias achieve acceptable recurrence rates, suggesting that expertise can mitigate complexity concerns [26].

Our multi-modal detection approach addressed known screening limitations. While validated tools like PINCH-Phone demonstrate 82% sensitivity, the 18% false-negative rate necessitated our supplementary clinical and imaging review [27]. Although we used common screening questions rather than validated instruments like HerQLes, our comprehensive protocol aimed to maximize detection accuracy [28]. The weighted median follow-up of 23.5 months, with >80% completion at 36 months, exceeds benchmarks from major hernia studies, where follow-up typically ranges 70–85% at 24 months [6,13,29].

## 5. Limitations

This study has several limitations that merit consideration. First, the retrospective design, despite prospective data collection and propensity matching, cannot eliminate all selection bias or unmeasured confounding. Surgeon preference for extraction site selection may reflect patient factors not captured in our dataset. Second, our single-center experience over 15 years may limit generalizability. Surgical techniques, suture materials, and perioperative protocols evolved during the study period. Notably, five patients (3.8%) in the midline group received Vicryl (Ethicon, Inc.) sutures, which current guidelines discourage for fascial closure due to rapid absorption. Third, hernia detection relied partially on telephone screening using common literature questions rather than validated instruments. While we supplemented with clinical examination and imaging review, subclinical hernias may have been missed. The 36-month median follow-up, though exceeding most series, captures only 74% of expected lifetime hernias. Fourth, sample size constraints yielded wide confidence intervals for some estimates, particularly in multivariate models. The 18 POVH events limited our ability to perform comprehensive subgroup analyses or validate our risk stratification model in an independent cohort. Fifth, we could not standardize certain technical factors across surgeons, including suture tension, tissue bite size, and implementation of small-bites technique. The STITCH trial results emerged midway through our study period, potentially influencing later outcomes [30]. Finally, we lacked data on postoperative functional outcomes, patient-reported quality of life metrics beyond hernia-specific symptoms, and economic impact. These patient-centered outcomes increasingly guide surgical decision-making. Additionally, we did not collect data on nutritional status, frailty indices, or the impact of enhanced recovery protocols, which may influence wound healing and hernia development.

### Future Directions and Clinical Implications

Future research should focus on multicenter randomized trials directly comparing extraction techniques. Studies should include Pfannenstiel incisions, which demonstrate excellent outcomes, with POVH rates of 0–1.3% [12,13,31]. Prospective validation of IAP-related risk factors and development of optimization protocols represent critical next steps. Long-term studies examining repair complexity for lateral versus midline hernias would help surgeons balance prevention against potential repair challenges.

Clinically, our results support considering paramedian extraction for laparoscopic right colectomy, particularly in high-risk patients. The identification of modifiable risk factors—chronic cough, constipation, and nocturia—presents opportunities for preoperative optimization. While we observed no complex repairs in our paramedian group, surgeons must acknowledge potential lateral hernia repair challenges when counseling patients. Alternative approaches like Pfannenstiel extraction merit consideration where anatomically feasible. Enhanced surveillance for patients with multiple risk factors appears prudent given the 38.5% POVH rate in this subgroup. Integration of these findings into surgical guidelines could standardize extraction site selection and improve patient outcomes.

## 6. Conclusions

This propensity-matched analysis suggests that paramedian extraction sites are associated with lower POVH incidence compared to midline approaches in laparoscopic right colectomy. The identification of modifiable risk factors, particularly conditions causing increased intra-abdominal pressure (chronic cough, constipation, nocturia), highlights opportunities for perioperative optimization. While these findings support the consideration of paramedian extraction, particularly in high-risk patients with IAP-related conditions, prospective randomized trials are needed to confirm these observations and establish optimal extraction site selection in minimally invasive colorectal surgery.

## Figures and Tables

**Table 1:** Baseline characteristics before propensity score matching.

Variable	Paramedian (n = 200)	Midline (n = 350)	*p*-Value	SMD
Demographics				
Age, years	65.2 ± 12.3	71.4 ± 10.8	<0.001	0.537
Female sex	116 (58.0)	164 (46.9)	0.012	0.223
BMI, kg/m^2^	26.8 ± 4.2	27.3 ± 4.5	0.195	0.114
Comorbidities				
ASA classification ≥ 3	64 (32.0)	168 (48.0)	<0.001	0.330
Hypertension	104 (52.0)	238 (68.0)	<0.001	0.331
Diabetes mellitus	44 (22.0)	112 (32.0)	0.012	0.225
Smoking history	32 (16.0)	84 (24.0)	0.026	0.201
IAP-Related Conditions				
Chronic cough	8 (4.0)	42 (12.0)	0.002	0.294
Constipation	12 (6.0)	56 (16.0)	<0.001	0.322
Nocturia/BPH	20 (10.0)	70 (20.0)	0.002	0.284
POVH Outcome				
Overall POVH incidence	4 (2.0)	54 (15.4)	<0.001	0.490

Data presented as n (%) or mean ± SD. SMD = standardized mean difference.

**Table 2:** Baseline characteristics after propensity score matching.

Variable	Paramedian (n = 133)	Midline (n = 133)	*p*-Value	SMD
Demographics				
Age, years	68.8 ± 11.9	69.2 ± 11.6	0.782	0.034
Age ≥ 65 years	97 (72.9)	99 (74.4)	0.788	0.033
Female sex	69 (51.9)	68 (51.1)	0.901	0.015
BMI, kg/m^2^	27.0 ± 4.3	27.1 ± 4.4	0.851	0.023
Comorbidities				
ASA classification ≥ 3	54 (40.6)	56 (42.1)	0.805	0.030
Hypertension	82 (61.7)	84 (63.2)	0.802	0.031
Diabetes mellitus	37 (27.8)	39 (29.3)	0.786	0.033
Coronary artery disease	23 (17.3)	25 (18.8)	0.751	0.039
Chronic renal failure	4 (3.0)	5 (3.8)	0.732	0.043
Liver disease	2 (1.5)	3 (2.3)	0.651	0.057
COPD	14 (10.5)	15 (11.3)	0.844	0.025
Smoking history	26 (19.5)	28 (21.1)	0.758	0.039
IAP-Related Conditions				
Chronic cough	10 (7.5)	11 (8.3)	0.820	0.028
Constipation	14 (10.5)	15 (11.3)	0.844	0.025
Nocturia/BPH	17 (12.8)	18 (13.5)	0.856	0.022
Surgical History				
Previous abdominal surgery	46 (34.6)	48 (36.1)	0.798	0.031
Previous hernia repair	8 (6.0)	9 (6.8)	0.804	0.031
Oncologic Variables				
Malignancy indication	118 (88.7)	119 (89.5)	0.848	0.024

Data presented as n (%) or mean ± SD. SMD = standardized mean difference; BMI = body mass index; ASA = American Society of Anesthesiologists; COPD = chronic obstructive pulmonary disease; IAP = intra-abdominal pressure; BPH = benign prostatic hyperplasia.

**Table 3:** Operative characteristics (after matching).

Variable	Paramedian (n = 133)	Midline (n = 133)	*p*-Value	SMD
Surgical Approach				
Laparoscopic completion	130 (97.7)	127 (95.5)	0.307	0.127
Conversion to open	3 (2.3)	6 (4.5)	0.307	0.127
Operative Details				
Operative time, min	142 ± 38	145 ± 41	0.540	0.075
Extended resection *	12 (9.0)	14 (10.5)	0.681	0.051
Intracorporeal anastomosis	26 (19.5)	24 (18.0)	0.756	0.038
Extraction Site Characteristics				
Incision orientation			<0.001	0.612
-Horizontal	21 (15.8)	0 (0.0)		
-Vertical	112 (84.2)	133 (100.0)		
Incision length, cm	5.8 ± 1.2	6.1 ± 1.4	0.062	0.231
Specimen diameter, cm	4.2 ± 1.8	4.3 ± 1.9	0.662	0.054
Technical Factors				
Wound protector use	133 (100.0)	127 (95.5)	0.029	0.309
Suture material			0.617	0.087
-PDS ^$^	126 (94.7)	128 (96.2)		
-Vicryl ^#^	7 (5.3)	5 (3.8)		
Skin closure			0.734	0.059
-Staples	89 (66.9)	91 (68.4)		
-Suture	44 (33.1)	42 (31.6)		

Data presented as n (%) or mean ± SD. SMD = standardized mean difference. * Extended resection defined as en bloc resection of adjacent organs or structures. ^#^ Vicryl (Ethicon, Inc., Somerville, NJ, USA); ^$^ PDS (Ethicon, Inc., Somerville, NJ, USA).

**Table 4:** Postoperative outcomes and follow-up (after matching).

Variable	Paramedian (n = 133)	Midline (n = 133)	*p*-Value	SMD
Postoperative Complications				
Clavien–Dindo ≥ III	3 (2.3)	4 (3.0)	1.000	0.045
Surgical site infection	4 (3.0)	5 (3.8)	1.000	0.042
Ileus	2 (1.5)	4 (3.0)	0.680	0.101
Postoperative pneumonia	1 (0.8)	1 (0.8)	1.000	<0.001
ARDS	0 (0.0)	0 (0.0)	NA	<0.001
ICU days	0.00 ± 0.00	0.12 ± 1.01	0.170	0.169
Length of stay, days	5.2 ± 2.1	5.4 ± 2.3	0.462	0.090
Readmission within 30 days	11 (8.3)	13 (9.8)	0.668	0.053
Follow-up Data				
Follow-up at 12 months	125 (94.0)	122 (91.7)	0.467	0.089
Follow-up at 24 months	117 (88.0)	114 (85.7)	0.589	0.066
Follow-up at 36 months	110 (82.7)	107 (80.5)	0.640	0.057
Median follow-up, months	34.5 (28–36)	35.1 (29–36)	0.707	0.046
POVH Outcomes				
**Overall POVH incidence**	**3 (2.3)**	**15 (11.3)**	**0.007**	**0.365**
POVH timing				
-6–12 months	0 (0.0)	4 (3.0)	0.131	0.249
-12–24 months	2 (1.5)	3 (2.3)	1.000	0.055
**-24–36 months**	**1 (0.8)**	**8 (6.0)**	**0.042**	**0.294**
POVH presentation				
-Asymptomatic (incidental)	1 (0.8)	4 (3.0)	0.367	0.167
**-Patient-reported symptoms**	**1 (0.8)**	**7 (5.3)**	**0.073**	**0.266**
**-Clinical diagnosis**	**1 (0.8)**	**7 (5.3)**	**0.073**	**0.266**
**Requiring surgical repair**	**1 (0.8)**	**7 (5.3)**	**0.073**	**0.266**
Quality of Life Impact (among POVH patients)				
-Minimal/None	1 (33.3)	5 (33.3)	1.000	<0.001
-Moderate	1 (33.3)	4 (26.7)	1.000	0.145
-Severe	1 (33.3)	2 (13.3)	0.470	0.483

Data presented as n (%), mean ± SD, or median (IQR). SMD = standardized mean difference. Bold indicates primary outcome with statistical significance.

**Table 5:** Univariate analysis of risk factors for POVH (n = 266).

Variable	No POVH (n = 248)	POVH (n = 18)	OR (95% CI)	*p*-Value
Surgical Factors				
Midline extraction	118 (47.6)	15 (83.3)	5.51 (1.76–24.2)	0.008
Open conversion	3 (1.2)	6 (33.3)	40.8 (9.62–213)	<0.001
Incision length (per cm)	5.8 ± 1.3	7.2 ± 1.8	1.95 (1.42–2.68)	<0.001
Intracorporeal anastomosis	50 (20.2)	0 (0.0)	-	0.999
Operative time (per 30 min)	143 ± 39	162 ± 48	1.31 (1.05–1.64)	0.018
Demographics				
Age (per year)	69.0 ± 11.7	68.6 ± 12.2	0.99 (0.96–1.04)	0.775
Female sex	130 (52.4)	7 (38.9)	0.72 (0.26–1.90)	0.519
BMI (per kg/m^2^)	27.0 ± 4.3	28.3 ± 4.8	1.08 (0.97–1.21)	0.182
IAP-Related Conditions				
Constipation	14 (5.6)	15 (83.3)	47.1 (15.0–163)	<0.001
Chronic cough	11 (4.4)	10 (55.6)	33.9 (11.3–110)	<0.001
Nocturia/BPH	24 (9.7)	11 (61.1)	25.7 (7.41–93.3)	<0.001
Smoking	43 (17.3)	11 (61.1)	8.22 (2.88–23.1)	<0.001
Medical Comorbidities				
ASA ≥ 3	97 (39.1)	13 (72.2)	4.01 (1.52–12.0)	0.007
Diabetes mellitus	71 (28.6)	5 (27.8)	1.58 (0.55–4.26)	0.380
Hypertension	151 (60.9)	15 (83.3)	1.89 (0.62–7.00)	0.283
COPD	24 (9.7)	5 (27.8)	3.48 (1.13–9.75)	0.026
Chronic renal failure	6 (2.4)	3 (16.7)	11.4 (2.23–50.9)	0.006
Liver disease	3 (1.2)	2 (11.1)	9.47 (1.13–55.1)	0.041
Surgical History				
Previous abdominal surgery	86 (34.7)	8 (44.4)	1.62 (0.61–4.26)	0.325
Previous hernia repair	14 (5.6)	3 (16.7)	3.59 (0.82–12.6)	0.087

Data presented as n (%) or mean ± SD. OR = odds ratio; CI = confidence interval; IAP = intra-abdominal pressure.

**Table 6:** Multivariate logistic regression models.

Variable	Model 1: Technical Factors	Model 2: Firth Penalized (Full)
	**aOR (95% CI)**	***p*-Value**
Primary Exposure		
Midline extraction	6.00 (1.68–31.3)	0.006
Surgical Factors		
Open conversion	45.6 (9.28–300)	<0.001
Incision length (per cm)	1.42 (0.91–2.21)	0.121
IAP-Related Conditions		
Chronic cough	-	-
Constipation	-	-
Nocturia/BPH	-	-
Smoking	-	-
Model Performance		
C-statistic	0.812	
Hosmer–Lemeshow *p* *	0.482	
AIC	128.4	
Likelihood ratio χ^2^	42.8	<0.001

aOR = adjusted odds ratio; CI = confidence interval; IAP = intra-abdominal pressure. Model 1 includes surgical factors only; Model 2 includes all significant univariate predictors. * Hosmer–Lemeshow test not applicable for Firth regression.

## Data Availability

The data presented in this study is available on request from the corresponding author. The data is not publicly available due to privacy and ethical restrictions.

## Supplementary Materials

The following supporting information can be downloaded at: https://www.mdpi.com/article/10.3390/jcm14155198/s1, Table S1: Baseline Characteristics Before Propensity Score Matching (all the data); Table S2: Baseline Characteristics of Patients Lost to Follow-up vs. Analyzed Cohort.

## Abbreviations

POVH—postoperative ventral hernia; BMI—body mass index; OR—odds ratio; CI—confidence interval; SMD—standardized mean difference; IAP—intra-abdominal pressure.

## Appendix A. Commonly Used Screening Questions and Associated Studies

These questions are frequently used in postoperative assessments to identify potential incisional hernias:

1. “Do you feel a bulge at or near your surgical incision?”
  ◦ Details: In a cohort study assessing patient-reported outcomes after incisional hernia repair, patients were asked about the presence of a bulge as an indication for surgical repair [28]
2. “Does the bulge increase with standing, coughing, or physical exertion?”
  ◦ The study included questions regarding the dynamic nature of the bulge, such as changes during physical activity, to assess hernia symptoms [28].
3. “Is there pain or discomfort at the site of your previous incision?”
  ◦ Patients reported postoperative symptoms, including pain and discomfort at the surgical site, which were used to evaluate the success of hernia repair [28]
4. “Has any physician diagnosed you with a hernia at the incision site?”
  ◦ The study collected data on clinical diagnoses of hernia recurrence to correlate with patient-reported symptoms [28].
5. “Have you undergone any imaging (e.g., ultrasound, CT) for abdominal wall concerns?”
  ◦ Information on imaging studies was gathered to support the assessment of hernia recurrence and validate patient-reported outcomes [28]
