# Supplementary material for: Comparison of Paramedian Versus Midline Extraction Sites in Elective Laparoscopic Right Colectomy: A Propensity-Matched Study of Postoperative Ventral Hernia Development
Source: J Clin Med. 2025 Jul 22;14(15):5198. doi: 10.3390/jcm14155198 (PMC12347929; doi:10.3390/jcm14155198)
Supplement: Supplementary file 1 [file jcm-14-05198-s001.zip › jcm-3703489-supplementary.pdf]

Supplementary Materials

Table S1. Baseline Characteristics Before Propensity Score Matching (all the data).

Variable	Paramedian (n=200)	Midline (n=350)	p-value	SMD
**Demographics**				
Age, years*	65.2 ± 12.3	71.4 ± 10.8	<0.001	0.537
Age ≥65 years*	124 (62.0)	273 (78.0)	<0.001	0.354
Female sex*	116 (58.0)	164 (46.9)	0.012	0.223
BMI, kg/m²*	26.8 ± 4.2	27.3 ± 4.5	0.195	0.114
BMI ≥30 kg/m²	48 (24.0)	105 (30.0)	0.132	0.134
**Comorbidities**				
ASA classification			<0.001	0.416
- ASA 1	28 (14.0)	21 (6.0)		
- ASA 2	108 (54.0)	161 (46.0)		
- ASA 3	60 (30.0)	154 (44.0)		
- ASA 4	4 (2.0)	14 (4.0)		
ASA ≥3*	64 (32.0)	168 (48.0)	<0.001	0.330
Hypertension*	104 (52.0)	238 (68.0)	<0.001	0.331
Diabetes mellitus*	44 (22.0)	112 (32.0)	0.012	0.225
Coronary artery disease*	28 (14.0)	77 (22.0)	0.022	0.208
Chronic renal failure*	4 (2.0)	21 (6.0)	0.031	0.206
Liver disease*	2 (1.0)	14 (4.0)	0.048	0.196
COPD*	16 (8.0)	49 (14.0)	0.036	0.192
Peripheral vascular disease	12 (6.0)	35 (10.0)	0.108	0.146
Smoking history*	32 (16.0)	84 (24.0)	0.026	0.201
**IAP-Related Conditions**				
Chronic cough*	8 (4.0)	42 (12.0)	0.002	0.294
Constipation*	12 (6.0)	56 (16.0)	<0.001	0.322
Nocturia/BPH*	20 (10.0)	70 (20.0)	0.002	0.284
Chronic steroid use	8 (4.0)	28 (8.0)	0.071	0.168
**Surgical History**				
Previous abdominal surgery*	56 (28.0)	133 (38.0)	0.017	0.213
Previous hernia repair*	10 (5.0)	28 (8.0)	0.181	0.122
Previous bowel resection	4 (2.0)	21 (6.0)	0.031	0.206
**Laboratory Values**				
Hemoglobin, g/dL	12.8 ± 1.9	12.2 ± 2.1	<0.001	0.299
Albumin <3.5 g/dL	16 (8.0)	56 (16.0)	0.008	0.247
Creatinine >1.5 mg/dL	12 (6.0)	42 (12.0)	0.024	0.212
**Surgeon Experience**				
Senior surgeon (>50 cases/year)	152 (76.0)	210 (60.0)	<0.001	0.348
**Year of Surgery**			0.018	0.284
- 2009-2012	44 (22.0)	112 (32.0)		
- 2013-2016	68 (34.0)	119 (34.0)		
- 2017-2020	56 (28.0)	77 (22.0)		
- 2021-2024	32 (16.0)	42 (12.0)		
**Primary Outcome**				
**POVH incidence**	**4 (2.0)**	**54 (15.4)**	**<0.001**	**0.490**
Time to POVH, months†	19.5 ± 8.2	22.1 ± 9.8	0.614	0.280

Data presented as n (%) or mean ± SD. SMD = standardized mean difference; BMI = body mass index; ASA = American Society of Anesthesiologists; COPD = chronic obstructive pulmonary disease; IAP = intra-abdominal pressure; BPH = benign prostatic hyperplasia. *Variables included in the propensity score model. †Among patients who developed POVH only. Bold indicates primary outcome with statistical significance.

Table S2. Baseline Characteristics of Patients Lost to Follow-up vs. Analyzed Cohort

Variable	Lost to Follow-up (n=42)	Analyzed Cohort (n=550)	p-value
Age, years	68.2 ± 11.5	69.3 ± 11.4	0.542
Female sex	20 (47.6%)	280 (50.9%)	0.681
BMI, kg/m²	26.9 ± 4.6	27.1 ± 4.4	0.774
ASA ≥3	17 (40.5%)	232 (42.2%)	0.829
Diabetes mellitus	11 (26.2%)	156 (28.4%)	0.761
Hypertension	25 (59.5%)	342 (62.2%)	0.731
Extraction site			0.856
- Paramedian	14 (33.3%)	200 (36.4%)	
- Midline	28 (66.7%)	350 (63.6%)	

Data presented as n (%) or mean ± SD. No significant differences were observed between groups (all p>0.05), indicating minimal selection bias. ASA = American Society of Anesthesiologists; BMI = body mass index.
